# The Story of the Red Cross

**Published:** 1917-12-01

**Authors:** Arthur Stanley

**Affiliations:** M.P., Chairman J.W.C.


### THE STORY OF THE RED CROSS.

### By the Hon. Sir Arthur Stanley, G.B.E., M.P., Chairman J.W.C.

A lecture on this subject was delivered at the Royal Institute of Public Health, on the 21st November, by the Hon. Sir Arthur Stanley, G.B.E., C.B., M.P. Sir John Hewett, the Chairman, reminded the meeting that the lecturer was the Chairman of the Joint War Committee. As one who had himself worked on that committee, it gave him the opportunity to bear public testimony to the extraordinary services which Sir Arthur had rendered to Red Cross work in every theatre of the war, whilst his indefatigable energy had enabled the committee to spend the *Times* fund, which now reached £8,000,000, to the best possible advantage of those for whom it had been contributed.

Sir Arthur Stanley said the fact that the British Red Cross Society and the Order of St. John were now working together was not to be accredited to him: it was due to the fact that all the members of the Joint War Committee were determined that the money contributed should be used in the best way for the relief of the sick and suffering men of the Services.

### The Position of The Red Cross in War.

His first difficulty in speaking on the Red Cross work in the war was that of defining exactly what that work was. Yet when the American Red Cross Society came to consult with them as to the best methods of procedure, that was the question asked—namely, what position a Red Cross Society ought to take up in a war. The best definition he had seen was that given in the "Life" of Lord Wantage, the pioneer of Red Cross work in England. In that work an account was given of the gradual formation of the National Aid Society, the forbear of the British Red Cross Society, and the author said that however well organised an Army Medical Service might be, it had never been, and never would be, able to cope adequately with the sudden emergencies of war on a large scale. He held that voluntary organisations, unimpeded by official restriction, were alone capable of giving auxiliary relief and of providing extra comforts and luxuries with the requisite promptitude and rapidity. He felt, moreover, that the British public would always insist on taking a personal share in alleviating the sufferings of their soldiers, and that some recognised and authorised channel through which public generosity might flow was a matter of paramount importance. At the outbreak of war, the Red Cross organisation was as little prepared for a great European war as was the Army itself. It was almost impossible to get people in this country to prepare for something which they felt convinced was not in the least likely to happen. When the first circulars were sent out in connection with the V.A.D. organisation connected with the Territorial Army, asking people what they were prepared to do in time of war, replies were received from all over the country to the effect that it was useless

### THE STORY OF THE RED CROSS—(*continued*).

to ask what would be done in an emergency which was not to be expected. The idea then was a possible invasion of this country. At the commencement of the war, the staff of the Red Cross Society consisted of a secretary and two clerks, and the money available when the war broke out was something like £7,000. It had been laid down when the British Red Cross Society was formed, soon after the Boer War, that that was the only channel through which voluntary help would be accepted by the War Office and the Admiralty in time of war. They found that there were many other people in the field, notably the Order of St. John, which had done splendid work, especially in the training of men. They were helped very largely in coming to an agreement with the Order of St. John by the fact that the *Times* had undertaken to raise a large sum of money—namely, £200,000—for the Red Cross Society. It was possible that after the *Times* had raised that sum further amounts might have been collected for the Order of St. John, and they would thus have found themselves in the position of raising funds against one another and spending them in rivalry. He thought they would agree with him that they had adopted the best course when they came together and agreed to have a Joint Committee, to pool their funds and spend them in conjunction with one another.

### The Promptitude of Red Cross Methods.

The R.A.M.C. could not cope with all the work required, and that was where the Red Cross Society was called in to help. In the early days they began with supplying stores which were neither comforts nor luxuries, but necessities. It was felt that the public would forgive them even if the subscribed money were not spent quite regularly. One day he was telling Sir Alfred Keogh, at the War Office, of the stores which the Red Cross organisation had been able to send out at short notice, and Sir Alfred said he had a request to make which would take that body all its time to carry out. There had arisen a sudden demand for 4,000 hot-water bottles, and they would be required within a week. He, Sir Arthur, went back to Pall Mall, and found that a man had been in the day before and given them 2,000 hot-water bottles, and the result was that the 4,000 hot-water bottles were sent off the next morning. The doctors and nurses available for the Red Cross Service were very much less numerous than now. On one occasion, in response to another sudden demand, a 100-bedded hospital was dispatched, with the necessary *personnel*, on the day the request was made. Here again it was sheer good luck, for that equipment had been meant for Ostend, and meantime that town had been taken by the Germans. These instances illustrated the usefulness of such an elastic body as this was.

### The Supply of Ambulances—Wonderful Developments.

Under the horse-ambulance conditions, which were operative in the first days of the war, the transfer of the wounded considerable distances in springless carts imposed a terrible amount of suffering. Later, permission was obtained to keep behind the fighting lines and search for missing and wounded, and any who might have strayed into village cottages. But the *Times* Fund soon made known the need for motor ambulances for transporting the sick and wounded, and then the public of England did as they always did when there was real need, subscribe generously. The result was that in a short time motor ambulances were supplied which completely revolutionised the whole system of the transport of sick and wounded. There were now about 2,000 motor ambulances in this Service, and not more than five different makes, so that the supply of spare parts had been much simplified. In the early days, of 200 cars sent abroad, there were some eighty-three different makes, each requiring its special fittings, and none were interchangeable.

### A Fleet of Motor Launches.

Another department was the provision of a fleet of motor launches for Mesopotamia, a request they never thought of being made. There were now forty or fifty of these at Bagdad alone, and the work they had done was absolutely inestimable. They had also been able to get over there a hospital ship, before the monsoon set in, a very important matter.

### The V.A.D. Organisation.

One of the most striking developments of Red Cross work in England itself was the V.A.D. organisation, and very few people knew exactly what it was. When the Territorial Army was created there was a gap in the hospital arrangements which required to be filled—*i.e.* the *personnel* of hospitals which would be set up in England in an emergency, such as at a time of invasion. The duty of filling that gap was bestowed on the Territorial Force Association, but there was a proviso that if they did not wish themselves to do the work, they might delegate it to the British Red Cross Society. Many counties did so delegate the work, but some did not. Out of that grew the Voluntary Aid Detachments, which were intended to be used not only if there should be an invasion, but as detachments to their own Commandant, to staff the hospitals in their own neighbourhood. As in other things, they soon had to break through the narrow bounds which were operative at the commencement, and V.A.D. hospitals grew up in many centres, and detachments in one district went to help in another. Not long after the beginning of the war there came a call for V.A.D. services in military hospitals at home, and afterwards for those abroad. There had been a most marvellous response, especially on the part of the women, who many times had received high praise from officers. The work of the responsible County Directors increased by being responsible for the issue of petrol, and for sugar control!

### The Star and Garter System.

Eighteen months ago it was brought to his notice that no provision existed for men who had become absolutely paralysed and disabled in the war. That seemed a legitimate use of money subscribed to the Red Cross Society, and out of the idea arose the Star and Garter System, that famous hotel having now become the centre from which radiated out efforts on behalf of these unfortunate men, and arrangements had been made for specialist and other skilled treatment. Worked, as it now was, in conjunction with the Pensions Ministry, there seemed no reason why any disabled man should not receive the treatment he required, and that branch of the science of medicine had recently made very great strides.

### Food for Prisoners of War.

Another branch of the Society's activities was that concerned with the sending of food to our men who were prisoners of war in Germany. At about the middle of 1916 the War Office and the Foreign Office began to be anxious about the amount of food which was being sent to these men by relatives and friends: some of them had fourteen or fifteen parcels sent to them in a fortnight, and it seemed clear that much of this food was being sold to the people of Germany. The V.A.D.s came to the rescue, and in a week or two procured statistics of what had been sent, and out of that a satisfactory system had been evolved.

